# A fully absorbable biomimetic polymeric micelle loaded with cisplatin as drug carrier for cancer therapy

**DOI:** 10.1093/rb/rbx012

**Published:** 2017-04-19

**Authors:** Weihua Zhuang, Boxuan Ma, Gongyan Liu, Xiaobing Chen, Yunbing Wang

**Affiliations:** 1 National Engineering Research Center for Biomaterials, Sichuan University, Chengdu 610064, China;; 2 Department of Biomass Chemistry, National Engineering Laboratory of Clean Technology of Leather Manufacture, Sichuan University, Chengdu 610064, China

**Keywords:** drug delivery, cisplatin, polymeric micelles, cancer therapy

## Abstract

cis-dichlorodiammineplatinum(II) (CDDP)-loaded polymeric micelles for cancer therapy have been developed to reduce the serious side effects of cisplatin CDDP. Herein, polymeric micelles incorporated with cisplatin are prepared based on the complexation between CDDP and hydrophilic poly (_L_-glutamic acid)-b-poly (2-methacryloyloxyethyl phosphorylcholine) (PLG-b-PMPC) diblock copolymers. These CDDP-loaded micelles possess an average size of 91 nm with narrow distribution, providing remarkable stability in media containing proteins. The release of CDDP from the micelles is faster at pH 5.0 and pH 6.0 than that at pH 7.4 and in a sustained manner without initial burst release. In addition, there is almost no difference in cellular uptake between these CDDP-loaded micelles and free CDDP. Moreover, in vitro cytotoxicity test shows they possess high efficacy to kill 4T1 cells as compared with free drug. Thus, PLG-b-PMPC copolymer might be a promising carrier for CDDP incorporating in cancer therapy.

## Introduction

Cisplatin is an important class of antitumor drug and has been widely used for the treatment of many cancers, such as non-small cell lung carcinoma [1], ovarian cancer [2], testicular cancer [3] and gastric cancer [4]. However, due to its severe dose-related side effects, i.e. neurotoxicity, nephrotoxicity, ototoxicity, nausea and myelosuppression [5–7], the clinical use of cisplatin is limited. Several cisplatin administration strategies and other platinum-based anti-tumor drugs have been used for reducing the disadvantages of cis-dichlorodiammineplatinum (II) (CDDP), which succeeded in less toxic but less efficacy [6–10]. Besides, the side effects are still the crucial barriers to increase dosage, which even result in irreversible renal failure [11]. Therefore, considerable efforts have been dedicated for delivery systems that target platinum drugs to solid tumors to reduce the side effects while improving the efficiency of the drugs.

Over the last two decades, the development of nanotechnology and the discovery of the enhanced permeability and retention (EPR) effect have provided powerful tools for drug delivery system (DDS) to reduce the side effects and improve the efficacy of antitumor drugs [12]. The EPR effect has been demonstrated to enhance the accumulation of long-circulating macromolecules, which has facilitated the application of drug-loaded nanocarriers [13, 14]. Moreover, nanocarriers with suitable particle size (1 0–100 nm) and narrow size distribution can avoid rapid renal clearance by reticuloendothelial system (RES) [15]. Nowadays, several kinds of CDDP-loaded nonocarriers, such as liposomes and polymer-platinum conjugates, have been develeped and even reached clinical trials [16]. However, these fomulations are still defective by poor stability, low loading efficacy, weak solubility or undesired aggregation behaviour [17]. To overcome these drawbacks, the strategies of assembling polymeric micelles by using block copolymers and cisplatin have been attractive to enhanced stability, superior blood half-life and tumor selectivity of CDDP-loaded nonocarriers [18–21]. Traditionally, polymers possess carboxyl groups have been investigated to incorporate cisplatin and form the hydrophobic core of micelles. Among them, polymeric micelles incorporating cisplatin prepared by Kataoka’s group via the polymer-metal complex formation between CDDP and poly(ethylene glycol)-b-poly (glutamic acid) block polymers have proceeded into clinical evalution [17, 22]. Although bioinspired poly (glutamic acid) with multi carboxyl groups is derived from naturally occurring glutamic acid, which is biocompatible and biodegradable [23].

For the above CDDP-loaded micelles, hydrophilic PEG is usually used as shell-forming block to protect the drugs inside the core, prevent the interaction with plasma proteins and cells, avoid the recognition by macrophages and prolong the circulation in the bloodstream [24–27]. Besides conventional PEG, zwitterionic polymers containing phosphorylcholine are also attractive for their excellent hydrophilic, biomimetic property, outstanding biocompatibility, high antithrombogenic properties and remarkable resistant to protein adsorption [28–30]. For instance, poly (2-methacryloyloxyethyl phosphorylcholine) (PMPC) has been introduced into several nanocarriers as shell-building blocks for drug delivery [31–35]. Due to its membrane-mimetic structure, PMPC has been reported to be helpful for DDS in facilitating cellular association and enhancing cellular uptake [35, 36]. In our previous work, we had synthesized a poly (γ-benzyl-_L_-glutamate)-block-poly (2-methacryloyloxyethyl phosphorylcholine) (PBLG-b-PMPC) copolymer for drug loading, which showed great antitumor efficacy [37]. However, the release of drugs was slow without stimuli-responsive property. Therefore, we hope to develop a more desirable drug carrier for quickly releasing the cargo in specific environment.

**Scheme 1.**
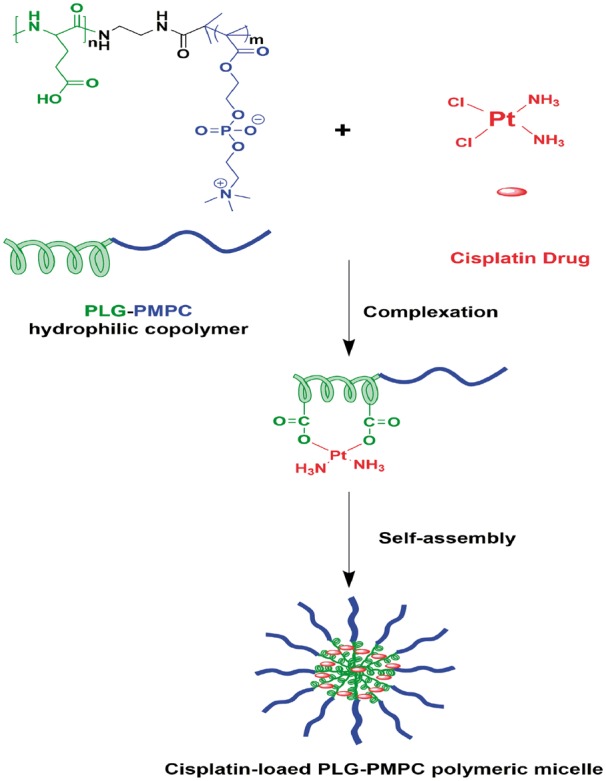
Schematic illustration of self-assembly of cisplatin-loaded PLG-b-PMPC polymeric micelles

In this work, hydrophilic poly (_L_-glutamic acid)-block-poly (2-methacryloyloxyethyl phosphorylcholine) (PLG-b-PMPC) copolymer is synthesized to complex with cisplatin, forming polymeric micelles as CDDP carriers through self-assembling (Scheme 1). The release of CDDP is controllable and shows a faster drug release at low pH. Additionally, these CDDP-loaded micelles possess an average size of sub-100 nm with narrow distribution, great protein stability, fast cellular uptake and also high tumor cells inhibition efficacy.

## Materials and methods

### Materials

γ-benzyl-_L_-glutamate-N-carboxyanhydride (BLG-NCA, 97%, Chengdu Enlai biological technology Co., Ltd, China) was recrystallized twice from n-hexane/ethyl acetate (1/1, V/V) before using. 2-methacryloyloxyethyl phosphorylcholine was purchased from Nanjing Natural Science and Technology Institute and used without further purification. N-Boc-ethylenediamine, 2-Bromoisobutyryl bromide, 3-(4, 5-Dimethyl-thiazol-2-yl)-2, 5-diphenyl tetrazolium bromide (MTT), and 2,2′-Dipyridyl (bpy) were purchased from Chengdu Best Reagent Co., LTD (Chengdu, China) and used as received. Cuprous bromide (CuBr) from Chengdu Best Reagent Co., LTD (Chengdu, China) was purified by washing with acetic acid and ethanol. Cisplatin (CDDP) was purchased from Shanghai Ziyi Reagent (Shanghai, China). Extra dry N, N-Dimethylformamide (DMF) was purchased from Arcos Organics. All other reagents and solvents were purchased from Chengdu KeLong Chemical Reagent Company (Chengdu, China) and used without further purification.

### Synthesis of PLG-b-PMPC copolymers

First, macroinitiator PBLG-Br was synthesized according to our previous report [37]. Then, PBLG-b-PMPC copolymers were synthesized by ATRP method. Briefly, PBLG-Br (1 g, 0.2 mmol) and MPC (2.1 g, 7.0 mmol) were dissolved in a dry flask with 30 ml solvent (MeOH/DMSO, 1/1, v/v). After the oxygen was removed by a freeze-pump-thaw procedure for three times, CuBr (28.8 mg, 0.2 mmol) and 2, 2′-dipyridyl (62.4 mg, 0.4 mmol) were added into the flask under the atmosphere of N_2_. The reaction was performed for 48 h at 38°C with flame-sealed and the protection of N_2_. The reaction mixture was purified by passing through a silica gel column, concentrated, and precipitated into excess ethyl ether. The product was dried in vacuum at room temperature overnight. For the deprotection of the benzyl groups, PBLG-b-PMPC was dissolved in 0.1 M NaOH at room temperature for 1 h. The mixture was dialyzed against deionized water for 2 days. PLG-b-PMPC diblock copolymer was obtained by precipitating into excess amount of cold diethyl ether and followed by vacuum drying.

### Preparation of CDDP-loaded polymeric micelles

Briefly, 20 mg PLG-b-PMPC and 4 mg CDDP were dissolved in 10 ml deionized water and stirred at 37°C for 72 h. CDDP-incorporated micelles thus prepared were purified by dialysis against deionized water for 24 h (MWCO 3500). The whole procedure was performed in the dark. The final volume of micellar solution was set to be 20 ml. The size distribution of the CDDP-loaded micelles was evaluated by a Malvern Zetasizer Nano ZS at 25°C. Drug loading content (DLC) and drug loading efficiency (DLE) were determined by inductively coupled plasma mass spectrometry (ICP-MS) and calculated by the following equations:

(1) $$DLC\left(\%\right)=\frac{mass\;of\;loaded\;CDDP}{mass\;of\;the\;CDDP-loaded\;micelles}\times 100\%$$

(2) $$DLE\left(\%\right)=\frac{mass\;of\;loaded\;CDDP}{mass\;of\;CDDP\;used\;for\;loading}\times 100\%$$

### *In vitro* drug release and micellar stability

The release profiles of CDDP from CDDP-loaded micelles were evaluated by dialysis method. Typically, 2 ml CDDP-loaded micelles solution (1 mg/ml) was add to a dialysis bag (MWCO 3500) in 100 ml PBS solution and then incubated at 37°C in the dark. At predetermined time intervals, 2 ml solution were taken out from the release media and 2 ml fresh PBS was added into the release media. The released platinum was confirmed by ICP-MS.

The stability of the micelles was evaluated by monitoring the particle size in 10, 20 and 50% (fetal bovine serum (FBS)/micellar solution, v/v) FBS over time.

### Cytotoxicity assay

The cytotoxicity of PLG-PMPC copolymers, free CDDP and CDDP-loaded PLG-b-PMPC micelles against 4T1 cells were evaluated by the MTT assay. 4T1 cells were seeded in 96-well plates at a density of 5000 cells per well in 200 μl RPMI 1640 culture medium, which contained 10% FBS and supplemented with penicillin (50 U/ml) and streptomycin (50 U/ml) and incubated at 37°C in 5% CO_2_ atmosphere for 24 h. Then the culture medium was replaced by 200 μl fresh medium, which contained varying concentration of PLG-PMPC, free CDDP and CDDP-loaded micelles. The cells were incubated for another 48 or 72 h. But for free PLG-b-PMPC, the cells were incubated for another 24 h. At each time point, 20 μl MTT solution (5 mg/ml) was added to each well and incubated for 4 h at 37°C. Then the medium was replaced by 200 μl DMSO, followed by shaking for 20 min. The cell viability was measured in a Bio-Rad 680 microplate reader at a wavelength of 490 nm. The relatively cell viability was calculated by the following formula:

(3) $$Cell\;viability\;\left(\%\right)=\frac{A_{sample}}{A_{blank}}\times 100\%$$

where, *A*_sample_ and *A*_blank_ referred to the absorbance of the sample wells and blank control wells, respectively. Data were presented as average ± SD (*n* = 3).

### Cellular uptake assay

4T1 cells were seeded in 24-well plates at a density of 5 × 10^4^ cells per well in 500 µl RPMI 1640 culture medium and cultured for 24 h. The original medium was replaced by 450 µL fresh medium and 50 µl CDDP-loaded micelles solution (the concentration of Pt was 0.1 mg/ml) or free CDDP solution with the same concentration of Pt. The cells were incubated for 2, 4 or 6 h at 37°C. At each selective time, the culture medium was suck away, then the cells were washed three times with PBS before being treated with aqua regia (HCl: HNO_3_ = 3: 1, volume ratio) for 4 h. The solution was diluted to determine the Pt concentration by ICP-MS.

### Characterization

^1^H NMR spectra were recorded on a spectrometer operating at 400 MHz (Bruker AMX-400). The polydispersity of PBLG-Br were determined by gel permeation chromatography (GPC) (Agilent 1260). The measurements were performed using DMF as the eluent at a flow rate of 1 ml/min at 40°C and a series of narrow PMMA standards for the calibration of the columns. The GPC measurement of PLG-b-PMPC diblock copolymer was conducted on a water GPC system (Agilent PL aquagel-OH column, EcoSEC (HLC-8320GPC with distilled water as eluent (25°C, flow rate: 1 ml/min, and polyethylene glycol as standards). Transmission electron microscopy (TEM) measurements were performed on a Hitachi H-600 transmission electron microscope with an accelerating voltage of 100 KV. A drop of the micelle solution (1 mg/ml) was deposited onto a 230 mesh copper grid and treated with 2% phosphotungstic acid at pH 6.5 negative stain solution, then allowed to dry at room temperature before measurements. Inductively coupled plasma mass spectrometry (ICP-MS, VG PQExCell, Thermo Jarrell Ash, USA) was used for determined the quantity of CDDP.

## Results and discussion

### Synthesis of hydrophilic PLG-b-PMPC copolymers

**Scheme 2.**
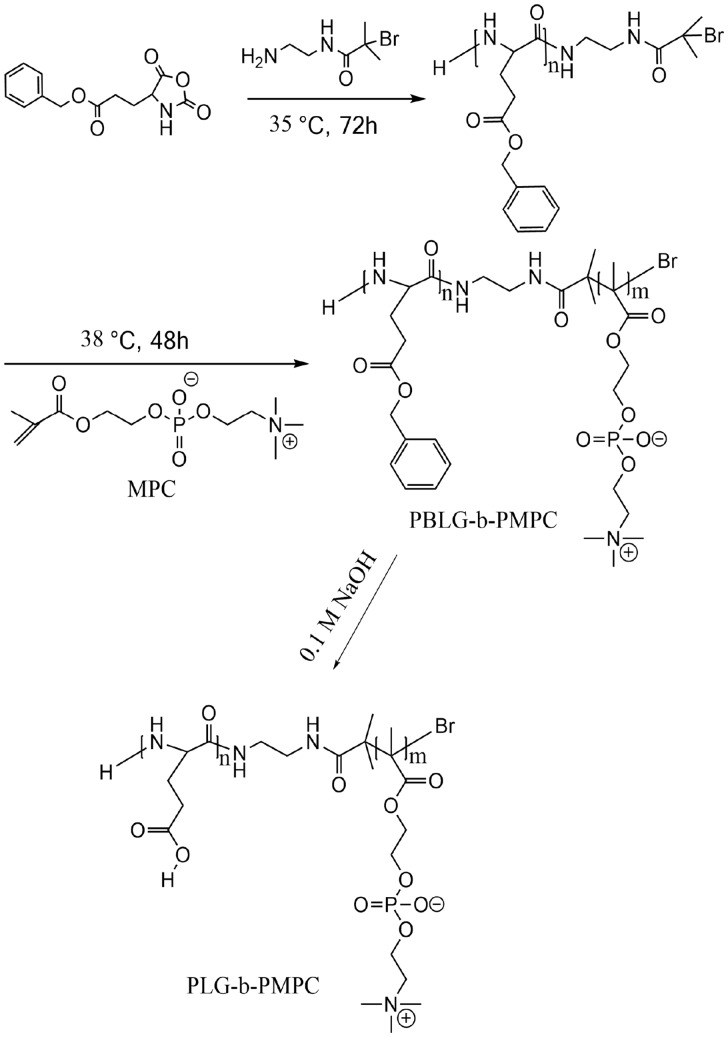
Synthetic route of PLG-b-PMPC

**Figure 1.**
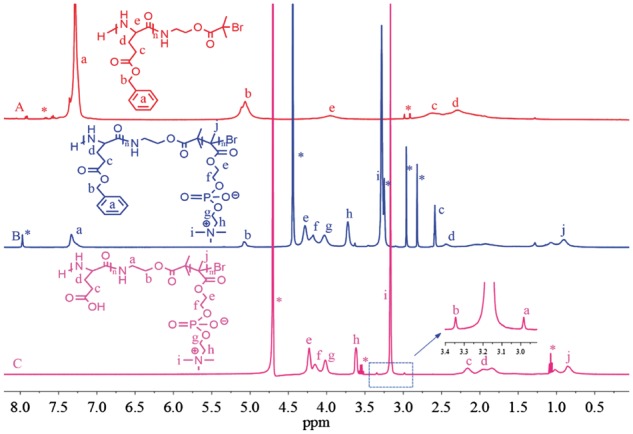
^1^H NMR Spectra of PBLG-Br **(A)** in DMSO-d_6_, PBLG-b-PMPC **(B)** in CD_3_OD and DMSO-d_6_ (1/1, V/V) and PLG-b-PMPC **(C)** in D_2_O

**Figure 2.**
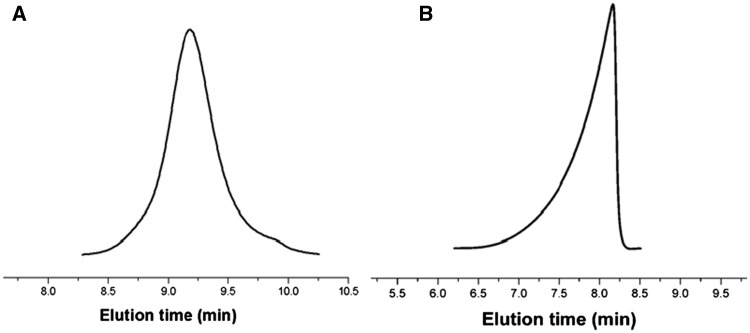
GPC Trace of PBLG-br macroinitiator in DMF **(A)** and PLG-b-PMPC diblock copolymer in water **(B)**

The synthetic route of PLG-b-PMPC was shown in Scheme 2. PBLG-Br macroinitiator was synthesized and its ^1^H NMR result was shown in Fig. 1A. The molecular weight distribution of PBLG-Br macroiniator was determined by GPC to be 1.22 (Fig. 2A). Amphiphilic PBLG-b-PMPC copolymers were obtained by ATRP of MPC using PBLG-Br as the initiator. The ^1^H NMR result of PBLG-b-PMPC was shown in Fig. 1B. When compared with Fig. 1A, characteristic peaks of PBLG (δ 7.34, –C_6_H_5_; δ 5.07, –CH_2_-C_6_H_5_) and characteristic peaks of PMPC (δ 4.27, -CH_2_-OC = O-; δ 4.17, -CH_2_CH_2_-OC = O-; δ 4.02, -CH_2_-CH_2_-N^+^(CH_3_)_3_; δ 3.71, -CH_2_-N^+^(CH_3_)_3_; δ 3.28, -N^+^(CH_3_)_3_) were found, suggesting the successful synthesis of PBLG-B-PMPC copolymer. Finally, hydrophilic PLG-b-PMPC copolymers were obtained by the deprotection of benzyl groups in NaOH solution. Obviously, Fig. 1C displayed that the benzyl group signals located at δ 7.34 ppm and methylene located at δ 5.07 ppm disappeared after the deprotection. Although the characteristic peaks of PLG-b-PMPC (δ 2.16, -CH_2_COOH; δ 1.96, -CH-CH_2_-; δ 4.23, -CH_2_-OC = O-; δ 4.15, -CH_2_CH2-OC = O-; δ 4.02, -CH_2_-CH2-N^+^(CH_3_)_3_; δ 3.61, -CH_2_-N^+^(CH_3_)_3_; δ 3.17, -N^+^(CH_3_)_3_) were found, indicating that the deprotection was complete. Furthermore, the DP of the PLG segment was calculated to be 21 based on the ratio of integration at peak b (-CH_2_-COO-, 2 H) and peak c (-CH_2_-COOH, 2 H) in Fig. 1C. Although the DP of the PMPC segment was calculated to be 34 based on the ratio of integration at peak b(-CH_2_-COO-, 2 H) and peak h (-CH_2_N^+^(CH_3_)_3_, 2 H). Therefore, the PLG-b-PMPC that we studied in this work can be defined as PLG_21_-b-PMPC_34_ with the molecular weight of about 12 700 g·mol^−^^1^ based on the ^1^H NMR result. The molecular weight of PLG-b-PMPC was further determined as 16 400 g•mol^−^^1^ by GPC with a PDI of 1.2 (Fig. 2B).

### Preparation and stability of CDDP-loaded PLG-b-PMPC polymeric micelles

**Figure 3.**
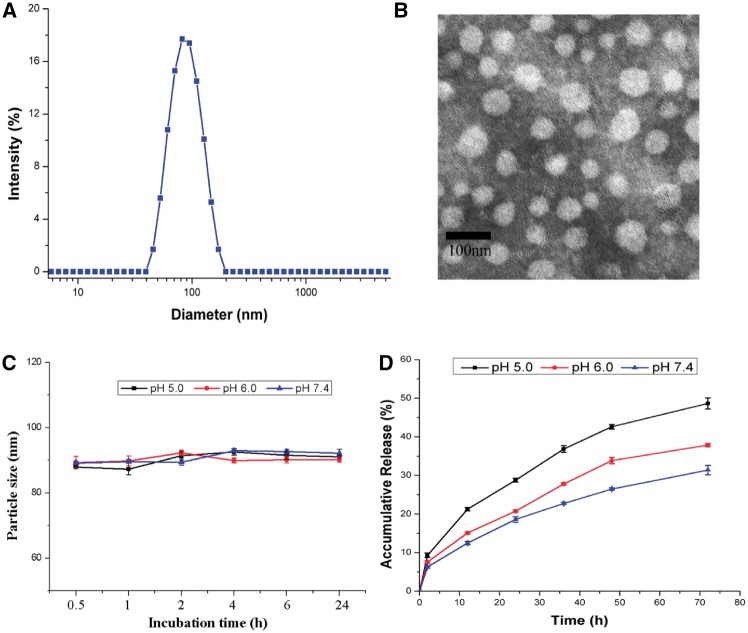
(A) The Size distribution of CDDP-loaded micelles with a PdI of 0.1 ± 0.04; **(B)** TEM image of CDDP-loaded micelles; **(C)** Particle size of CDDP-loaded micelles incubation with differents concentrations of FBS (FBS/micellar solution, v/v) at 37 °C as measured by DLS. The data were presented as the average ± SD (*n* = 3); **(D)** Accumulative release of CDDP-loaded micelles in PBS (pH = 5.0, 6.0 and 7.4). All measurements were carried out in triplicate. The data were presented as the average ± SD (*n* = 3)

As shown in Scheme 1, CDDP-loaded PLG-b-PMPC polymeric micelles could be prepared by complexing cisplatin with carboxylate groups of the PLG segment. The average size of these CDDP-incorporated micelles was measured by DLS, which was 91 nm with a narrow distribution of 0.10 ± 0.04 (Fig. 3A). In the TEM image, the result revealed the well formation of these CDDP-loaded micelles (Fig. 3B). The average diameter of the micelles in TEM image was around 43 nm which is smaller than that scaled by DLS, which might due to the dehydration of the PMPC shell during the sample preparation process for TEM. The DLC and drug loading efficacy (DLE) of Pt determined by ICP-MS were 14.8 and 86.5%, respectively, based on the CDDP/polymer feed ratio of 20%.

It was well known that the protein adhesion onto the drug carrier surface was one of the most important biological barriers that not only induced undesirable aggregation but also caused recognition by RES system, in which drug carriers would be rapidly cleared out of blood circulation [38]. In this study, one of the purpose to introduce biomimetic PMPC as micelle shell was to enhance the protein resistence of the DDS due to its zwitterionic structure. To investigate the stability of CDDP-loaded micelles, micellar solution (1 mg/ml) was incubated with 10, 20 and 50% FBS for up to 24 h, and the particle sizes were monitored by DLS. As shown in Fig. 3C, there was almost no change in the particle size of CDDP-loaded micelles with 10% FBS. Meanwhile, the particle size of CDDP-loaded micelles solution with 20 or 50% FBS only had a slight change, which indicated the great stability of CDDP-loaded micelles in biological solution.

### *In vitro* release behavior of CDDP from CDDP-loaded PLG-b-PMPC micelles

The *in vitro* release behavior of CDDP from CDDP-loaded PLG-b-PMPC micelles was investigated in PBS with pH 7.4, 6.0 and 5.0, respectively. It has been reported that CDDP-incorporated micelles could dissociate into unimers with CDDP release in chloride ion-rich solutions due to an inverse ligand substitution of the Pt (II) atom from the PLG segments to chloride. Moreover, cisplatin was usually released faster from these micelles under acidic condition than that at physiological condition due to the protonation of the carboxylic groups [22]. As shown in Fig. 3D, cisplatin was released in a sustained manner without initial burst release and the release of cisplatin at pH 5.0 and pH 6.0 were faster than that at pH 7.4, which was beneficial for drug release specifically in tumor tissue with acidic enviroment.

### Cellular uptake and cytotoxicity of CDDP-loaded PLG-b-PMPC micelles

**Figure 4.**
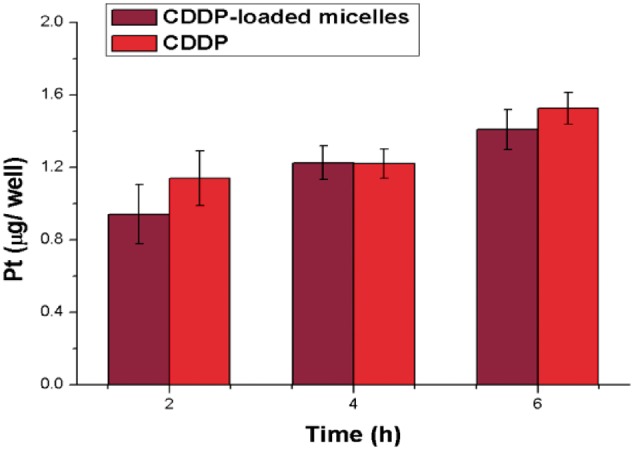
4T1 Uptake of free CDDP and CDDP-loaded micelles with the same concentration of pt. The data were presented as the average ± SD (*n* = 5)

The cellular uptake of CDDP-loaded PLG-b-PMPC micelles was quantified by ICP-MS with high detection sensitivity, and free CDDP was used as control. As shown in Fig. 4, the cellular uptake of the CDDP-loaded micelles was similar with free CDDP within 6 h, suggesting the fast cell internalization of these micelles. The similar phenomenon might be attributed to the biomimetic PMPC shell which was similar to the lipid bilayer and made it easily to be internalized by tumor cells, which was similar to with the work reported by Li’s group [34].

**Figure 5.**
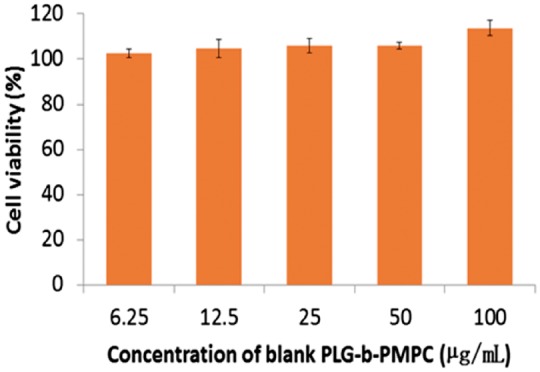
Cell viability of 4T1 cells incubated with blank PLG-b-PMPC at various concentration. The data were presented as the average ± SD (*n* = 3)

**Figure 6.**
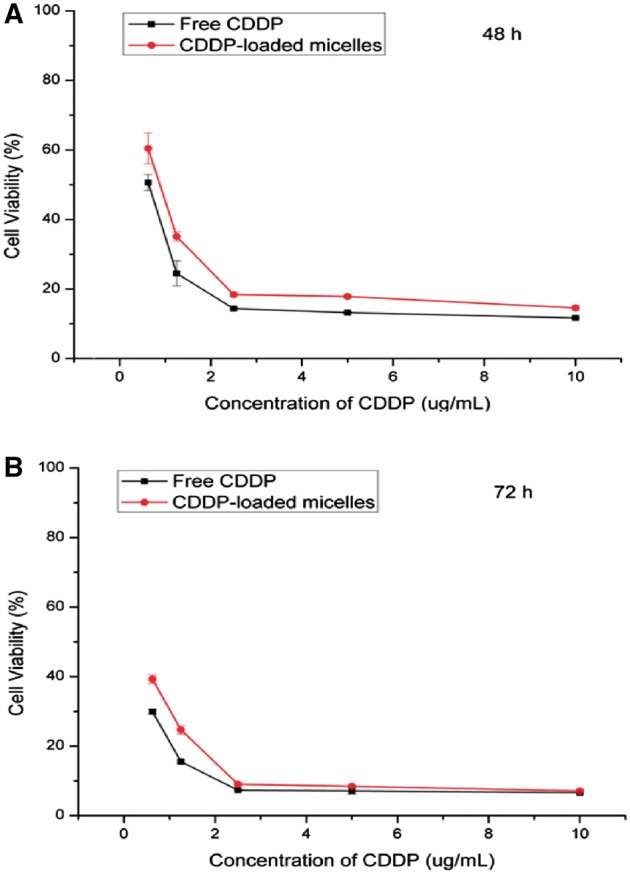
In vitro cytotoxicity of CDDP-loaded micelles and free CDDP against 4T1 cells after 48 h **(A)** and 72 h **(B)**. The data were presented as the average ± SD (*n* = 3)

The potential toxicity of PLG-b-PMPC block copolymer was first evaluated by MTT method against 4T1 cells. As shown in Fig. 5, the cell viability of 4T1 cells incubated with various concentration of PLG-b-PMPC was observed to be around 100% at all tests even at high concentration (100 µg/ml), indicating the excellent biocompatibility of PLG-b-PMPC. The great biocompatibility could be attributed to the zwitterionic PMPC shells of the micelles. The tumor cells inhibition efficacy of various concentrations of CDDP-loaded micelles was further evaluated against 4T1 cells after 48 and 72 h incubation, and free CDDP was used as control. As shown in Fig. 6, CDDP-loaded PLG-b-PMPC micelles exhibited similar inhibition efficiency with that of free CDDP at the same drug concentration after 48 and 72 h incubation. The result was consistent with the fast cell uptake behavior of the micelles (Fig. 4).

## Conclusions

In this work, a CDDP loaded biomometic nanocarrier was prepared by complexation between CDDP and the carboxyl groups of hydrophilic PLG-b-PMPC copolymer with a desirable particle size of 91 nm and narrow size distribution. These CDDP-loaded polymeric micelles with the zwitterionic surface exhibited excellent stability in media containing proteins. CDDP was released from micelles in a sustained manner without the initial burst release and the drug release was faster in acidic condition due to the protonation of the carboxylic groups. Moreover, these biomimetic CDDP-loaded micelles exhibited similar cell uptake and tumor cells inhibition efficacy as free CDDP, indicating a promising micellar formulation incorporating CDDP for cancer therapy.

## Funding

This research was supported by National Natural Science Foundation of China (Projects 51403131), Sichuan province Science-Technology Support Plan Project (2016SZ0004), the Programme of Introducing Talents of Discipline to Universities (B16033), and China Postdoctoral Science Foundation Funded Project (2015M570783).

*Conflict of interest statement*. None declared.
